# The Advantages of Robotic Over Open Thyroidectomy in Thyroid Diseases: A Systematic Review

**DOI:** 10.7759/cureus.26320

**Published:** 2022-06-25

**Authors:** Nathalie Haidar Ismail, Pardis Tavalla, Pulkita Uppal, Shaza Adel Awad mohammed, Shriya Rajashekar, Suganya Giri Ravindran, Meghana Kakarla, Musa Ausaja Gambo, Mustafa Yousri Salama, Pousette Hamid

**Affiliations:** 1 Research, California Institute of Behavioral Neurosciences & Psychology, California, USA; 2 Neurology, California Institute of Behavioral Neurosciences & Psychology, California, USA

**Keywords:** complications, thyroid diseases, advantages and disadvantages, open thyroidectomy, robotic thyroidectomy

## Abstract

Over a hundred thousand thyroid surgeries are performed per year in the United States. Although conventional thyroidectomy has successful surgical outcomes, robotic minimally invasive procedures, known for their scar free (regarding the neck, no collar incision) surgical outcomes gained popularity through the years. Furthermore, these techniques are new and still debatable. The purpose is to know the advantages of robotic over open thyroidectomy in thyroid diseases. Note that we didn't aim to compare different robotic techniques due to the lack of data.

We performed a systematic review comparing surgical approaches for thyroidectomy, open vs robotic techniques, from January 2017 to December 2021, according to the Preferred Reporting Items for Systematic Reviews and Meta-Analyses (PRISMA) 2020 guidelines. All papers with no full free article access and not in the English language were excluded. The outcomes of interest were superior cosmetics outcome, cost-effectiveness, limitations, operation time, length of hospital stay and postoperative pain or complications, and future outcomes.

A literature search was carried out in electronic databases (PubMed, Google Scholar) in order to retrieve all papers comparing the effectiveness of robotic vs open thyroidectomy. An initial reference search yielded 433 articles. Finally, we chose nine studies covering different robotic thyroidectomy techniques compared to the open thyroidectomy approach. Promising results were seen in these studies, especially with superior cosmetic results, less post-operative pain, swallowing discomfort, and voice changes. In addition, the risk of recurrent laryngeal nerve injury is almost the same as the open approach. Multiple types of biases were caused by the selection of the population and the limitation of the studies to certain regions associated with the low numbers of robotic thyroidectomy approaches in Europe and the United States of America and the lack of randomized trials and long-term follow-up respectively. All studies discussed the importance of the surgeon's skills and the patient decision in choosing the appropriate approach for the thyroidectomy depending on the risk factors, a larger number of patients, and longer follow-up from multiple hospitals.

## Introduction and background

The incidence of thyroidectomy worldwide has increased and in a world that gives a lot of importance to the physical appearance, the patients undergoing an open thyroidectomy, especially at a young age, are concerned about the anterior cervical incision [1]. Therefore, the robotic thyroidectomy approach has become more popular due to its post-operative scarless cosmetic result.

Lobe and colleagues performed the first robotic-assisted trans-axillary thyroidectomy (RATT) in 2005 [2,3]. The first procedure, done by Da Vinci robotic system (Intuitive Surgical, Sunnyvale, California), was performed by Chung in 2007 [4]. Then, in order to avoid some complications of the latter technique, Terris described the retro-auricular robotic thyroidectomy (RART) [4]. In addition, we discussed the bilateral axillary-breast robotic thyroidectomy (BABRT) [5,6], and the latest robotic technique, the transoral robotic thyroidectomy (TORT), was first reported in 2009 [1].

In this systematic review, we compared the advantages and inconveniences of all robotic thyroidectomy approaches to the conventional open thyroidectomy procedure. And we noticed that, although the robotic thyroidectomy is a minimally invasive procedure with satisfying cosmetic results, its direction in the future is still debatable. Most robotic thyroidectomy techniques consist of a Da Vinci robotic system (Intuitive Surgical, Sunnyvale, California) with a three-dimensional vision giving a real-time visualization and analysis of tissue perfusion along with recurrent nerve identification and sparing [3]. In addition, a tremor filtration software contributes to the decrease in the operator’s tremors.

Despite the limitations, many common advantages were noted in different robotic thyroidectomy over the open approaches [7]. For example, less post-operative pain, better swallowing, and more satisfactory cosmetic results. In addition, some differences exist within these robotic techniques, as due to its midline symmetrical exposure, the trans-axillary approach has better feasibility of lateral lymph nodes neck dissection than the other approaches, but on the other side, due to its large dissection flap, post-operative chest paresthesia may be experienced. This complication is evitable in the retro-auricular approach regarding its lower marge of dissection [4], resulting in a faster recovery and less post-operative discomfort compared to the trans-axillary thyroidectomy [7]. In addition, as per the American Thyroid Association (ATA), retro-auricular robotic thyroidectomy is feasible in obese patients contrary to the other robotic thyroidectomy techniques [8]. The disadvantage of these two robotic thyroidectomy techniques is the risk of brachial plexus injury, which is not present in the open thyroidectomy and can be prevented by repositioning the arm in an overhead flexed position [5-7]. Also regarding the latest robotic approach described, the trans-oral thyroidectomy, studies showed the necessity of antibiotics due to the buccal mucosal port of entrance, due to its access through the floor of the mouth and oral vestibule with the exposure to salivary bacteria, as opposed to this, to the open thyroidectomy and other robotic thyroidectomy approaches are considered as clean surgeries [4]. In addition, there can be an inability to control the hemorrhage in the trans-oral approach and the necessity of conversion to open thyroidectomy [1], but the advantage over the other robotic techniques and open thyroidectomy, is that it requires less dissection and without a post-operative collar scar [4].

## Review

Method

**Figure 1:**
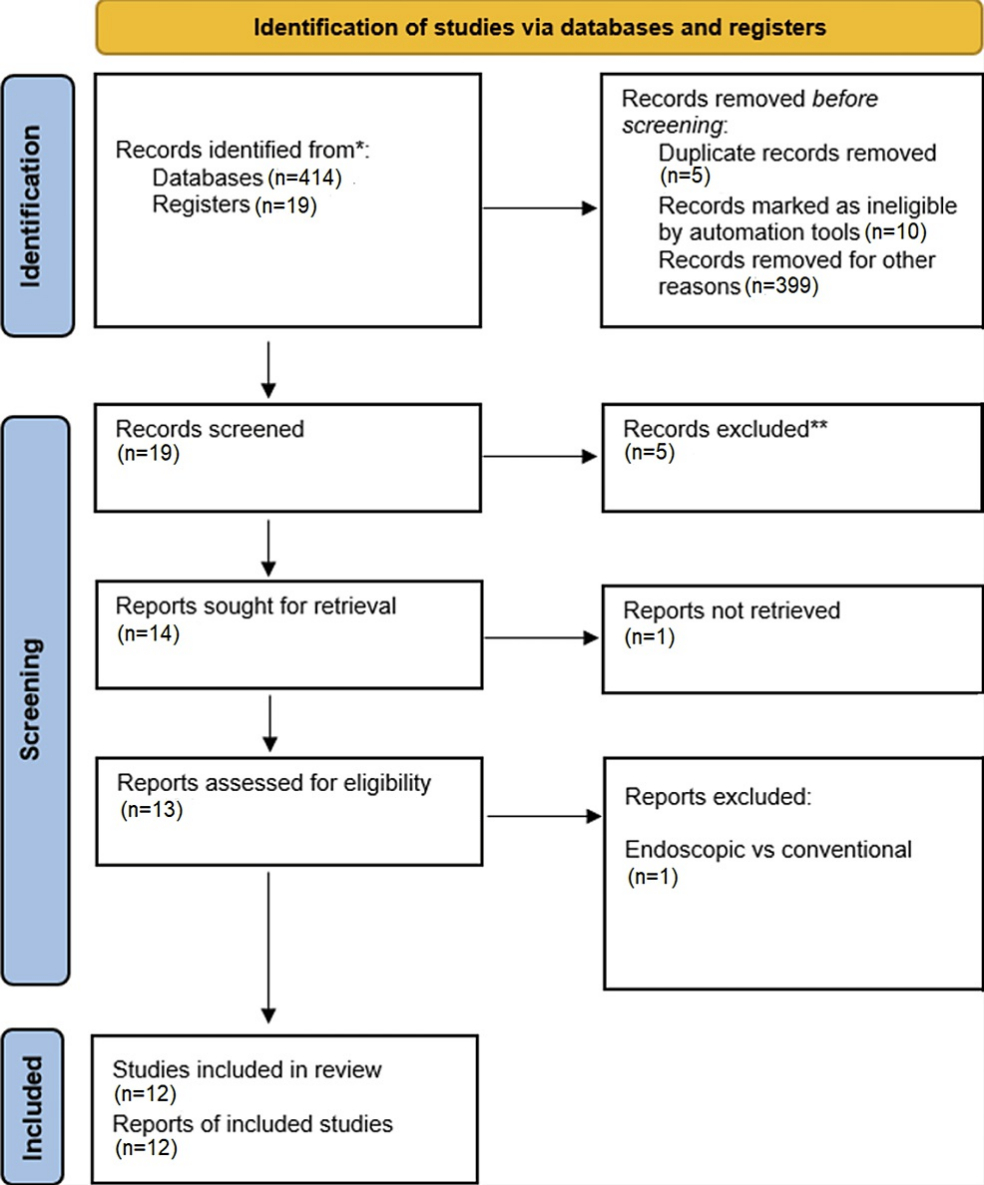
PRISMA flow diagram. PRISMA, Preferred Reporting Items for Systematic Reviews and Meta-analyses http://www.prisma-statement.org/

This is a systematic review performed according to the Preferred Reporting Items for Systematic Review and Meta-analysis Protocols, PRISMA 2020 checklist [6], as shown in Figure 1.

Search strategy

We performed a systematic literature search from January 2017 to December 2021 using PubMed and Google scholar for relevant full articles published in the English language. We analyzed patients with thyroid diseases/cancer who underwent open thyroidectomy or any approach of robotic thyroidectomy. The search strategy for PubMed, based on Medical Subject Headings (MESH) technique, is shown in Table 1.

**Table 1:** Search strategy for PubMed

Query	Search terms
#1	Robotic thyroidectomy OR robotic surgery OR robotic thyroid approach
#2	Thyroid diseases OR thyroid cancer
#3	Open thyroidectomy OR thyroid surgery
#4	#1 AND #2 AND #3

Study selection

Review articles and mini-review studies were used for the qualitative and quantitative synthesis of this systematic review. There is a lack of retrospective and cohort studies regarding this subject.

Eligibility criteria

Included studies were selected according to the following eligibility criteria: (a) Population - patients with thyroid diseases/cancer, all ages, TNM Classification of Malignant Tumors stage < 4, no previous neck surgery or radiotherapy; (b) Intervention - most popular techniques of robotic thyroidectomy (Trans-axillary, Retro-auricular, Bilateral axillary-breast, and Transoral); (c) Comparator - open thyroidectomy (OT); (d) Outcomes - costs, conversion, operation time, length of hospital stay and postoperative complications (pain, swallowing discomfort, voice changes, chest paresthesia), cosmetic outcomes.

Exclusion criteria: technical reports, books and documents, animal studies, and other nonrelevant studies were excluded from the analysis. Cases of neck surgery, radiation, or lateral neck disease were also excluded.

Data extraction

A standardized recording characteristic was used for data extraction, we started with an interval of five years of publication, from January 2017 to December 2021. As there weren’t enough studies, especially systematic reviews, comparing robotic and open approaches for thyroidectomy, we were limited in our study, that’s why we didn’t specify the country of origin or the gender and number of study participants. Regardless, we took different study outcomes into consideration. In our systematic study, we found a total of 12 studies, 11 were review articles and only one was a meta-analysis study. There were no gender preferences or age range, some studies showed a correlation between the patient weight and the difficulty of performing a robotic thyroidectomy approach and considered a BMI less than 25 kg/m² and nodule size less than three centimeters [2]. Operative outcomes and postoperative complications were also considered to be essential comparative characteristics between the robotic and the open thyroidectomy technique.

Methodological quality assessment

We evaluated the risk of bias using the assessment of multiple systematic reviews (AMSTAR) checklist for the meta-analysis study [6] and the study showed a low risk of bias. And the Scale for the quality Assessment of Narrative Review Articles (SANRA scale) for the eight remaining article reviews of our study (Table 2).

**Table 2:** Risk of bias assessment. SANRA* *Scale for the quality Assessment of Narrative Review Articles Richmon and Kim [1]; Aidan et al. [8]; Pavlidis et al. [9]; Chang et al. [4]; Alzahrani et al. [3]; Liu and Kim [5]; Kaliszewski et al. [7]; Fregoli et al. [10]; You et al. (Gland Surg., 2021) [11]; You et al. (surgery, 2021) [12]

	Justification of the article’s importance for the readership	Statement of concrete aims or formulation of questions	Description of the literature search	Referencing	Scientific reasoning	Appropriate presentation of data
Richmon and Kim	1	2	1	1	1	1
Aidan et al.	2	2	1	2	1	2
Pavlidis et al.	2	2	1	2	2	1
Chang et al.	2	2	1	2	2	2
Alzahrani et al.	2	2	1	2	2	0
Liuand Kim	2	2	1	2	2	2
Kaliszewski et al.	1	2	1	1	1	1
Fregoli et al.	2	2	2	2	1	1
You et al. (Gland Surg., 2021)	2	2	2	2	1	2
You et al. (Surgery, 2021)	2	1	2	2	1	1

Use of two different checklists aimed to judge each article by the rate of the bias’s risk. A study was considered to be of high quality when the risk of bias was rated as low or moderate. On the contrary, a study was considered to be of low quality, when the risk of bias was rated high.

Results

Literature Search

A total of 433 citations were found and only 12 reviews were included in this systematic review due to their high quality and also lack of studies regarding our subject. As we noticed from our study, robotic thyroidectomy techniques are mainly performed in the far east and there are still limitations applied by the ATA in the allowance of these techniques. 

Characteristics of the Included Studies

The studies included in this review were published from January 2017 to December 2021, among which eleven were review articles and one meta-analysis.

Patient Characteristics

A different range of ages with no gender preferences was considered. But we noticed that the number of female patients was higher than the male patients and this was explained by the physical and aesthetic concerns of the females compared with the males [1-6].

Surgical Outcome

Details about the procedure and the associated risks must be explained to all the patients and mentioned in an informed consent [3]. All of the articles in our study explained many advantages of the robotic thyroidectomy approach compared to the open technique. Furthermore, the Da Vinci Robotic device, with its three-dimensional real-time visualization, makes the robotic approach feasible and safe [2-3]. In addition, the post-operative complications such as pain [4-6], swallowing discomfort and cosmetic outcome results, and voice changes are better with the robotic technique [2,3]. 

Despite certain advantages of the robotic thyroidectomy, many limitations are present such as the operation duration which is shown to be two to three times more than the open technique [2], but the studies showed that with an increase in the surgeon’s learning curve and skills the operative time length decreases [6]. Also, some other limitations concern the body weight of the patient, the size of the lobe, and the nodule [2]. Moreover, the brachial plexus injuries and chest paresthesia due to the intra-operative positioning are avoided by placing the arm in flexed overhead position instead of overextension [3].

Risk of bias assessment

Most studies had a moderate risk of bias. Regional bias where more robotic thyroidectomies are performed in the far east and rarely in Europe and the United States of America [1-8]. According to the ATA, these techniques must be performed in high-volume centers where surgeons are skilled [8]. Selection bias is due to the elevated number of female patients [1-4] compared to the male patients. This can be explained by the importance of the physical appearance and esthetics of the female gender [5-9]. In addition to the guidelines reported by the ATA for patient selection according to multiple factors, such as the body weight index (≤25 kg/m²) [5] and patients not requiring lymph nodes dissection [8]. It also depends on the patient decision. In addition, the lack of studies and research centers that aim to assess a gold standard method for surgical resection.

Discussion

This systematic review is the first in comparing the robotic thyroidectomy technique in general, with the conventional one and concludes the overall result. All Robotic approaches apply the Da Vinci robotic system (Intuitive Surgical Inc., Sunnyvale, CA) which, as agreed in all the studies included in our systematic review, has an advantage over the open thyroidectomy considering the real-time visualization due to a three-dimensional view capable of identifying the tissue perfusion and the recurrent laryngeal nerve, avoiding its injury. In addition, there is a decrease in the operative tremor. All robotic techniques had a risk of brachial plexus injury due to the position of the ipsilateral arm by which this risk was reduced when the arm was placed in a flexed overhead position. First, we took each robotic technique study separately and then compared it to the open technique, before comparing the overall result. 

Robotic-assisted trans-axillary thyroidectomy (RATT) is the most performed gasless surgery [8] and is considered safe and feasible, but in contrast to the other robotic techniques, this approach consists of a flap creation superficial to the pectoralis muscle with resulting post-operative chest paresthesia [9]. In addition, several studies show reduced post-operative complications, including swallowing discomfort, pain, and satisfaction in cosmetic outcomes in comparison to the open approach [10].

Bilateral axillary-breast robotic thyroidectomy (BABRT) was first applied in 2007 [7-11]. Unlike other robotic techniques, it has a symmetrical approach to both thyroid lobes due to its midline accessibility, but also a skin flap dissection that is predisposed to post-operative chest paresthesia for all ages with a preference below 70 years of age due to the limitations resulting from high comorbidities. Eleven studies were conducted comparing the BABRT to the OT ( 1070: 1663 ) [6]. Nine studies showed an operative time almost 2.5 times longer in BABRT than OT (p<0.00001) and no remarkable difference between the two techniques regarding the tumor size ( p=0.11) [6] nor the length of hospital stay. The study also noted that the patients of BABRT were younger than the OT group ( p= 0.0003). Furthermore, three studies showed equivalence in post-operative pain for both techniques. Also, 10 studies noted no significant difference in recurrent laryngeal nerve (RLN) injury rates (0.25 <p<1.00) and a superior cosmetic objective satisfaction for the BABRT group. A small sample of patients showed no difference in central lymph nodes retrieval for both techniques but more studies are required to have more accurate results. In addition, there is a significantly high cost of BABRT compared with OT ( p<0.00001) [6]. Retro-auricular robotic thyroidectomy (RART) is characterized by a reduced range of dissection decreasing in that way to the esophagus and anterior chest vessels and nerves but with a high risk of marginal mandibular and greater auricular nerves injury. Transoral robotic thyroidectomy (TORT) is the newest approach [8] consisting of of carbon dioxide gas insufflation. It was first performed in 2015 on four human patients [1], where three of them experienced a post-operative temporary mental nerve paresthesia that was avoided by changing the positions of lateral ports. In addition, a study of 17 patients was done in two different hospitals, where all the patients were female and of the same average age, which showed a feasible and safe central lymph nodes dissection that was successively performed in 13 cases with scarless post-operative results and a mean operative time of 254 minutes [1], and a mean nodule size of 1.2 centimeters. Due to the recent engagement of this technique, there is a lack of comparative studies with the conventional open approach, the reason why multiple studies on a large number of patients with a long follow-up are needed [12]. 

In addition, robotic thyroidectomy is a feasible and safe technique with the three-dimensional and real-time visualization allowing a better analysis of tissue perfusion, also a decrease of the operator tremors due to a tremor infiltrative software [4]. Furthermore, regarding its oncological value, five years of follow-up for locoregional recurrence of thyroid cancer were similar (1.2% vs 1.2%) for both robotic and open approaches [4]. Although all the included studies showed a longer operative time for all types of robotic thyroidectomy, with an increase of three to four folds compared with the conventional open thyroidectomy, less post-operative pain, swallowing discomfort [1-4], and paresthesia at the site of the surgical incision are considered an improvement over the open approach [6]. In addition to the far east studies, many studies performed in the United Kingdom, Europe and North America, although small in number, agreed on the safety and the feasibility of all robotic thyroidectomy techniques [9]. Brachial plexus injuries and chest paresthesia for trans-axillary are avoided by placing the arm in flexed overhead position instead of overextension [4]. All robotic thyroidectomy approaches consist of a three-dimensional view [4-5] with a more clear visualization of tissue perfusion and identification of recurrent laryngeal nerve [1-2]. As a result, the risk of injury is almost the same, slightly less than the open technique [6-9].

The ATA reported that robotic thyroidectomy techniques might only be performed in high-volume centers with strict guidelines for patient selection. Also a contraindication to lateral neck dissection [8], one study suggested some pre-operatory imaging in order to identify the presence of lymph nodes according to which the appropriate procedure can be planned [1]. But it’s not a test applied systematically by the specified centers. Despite the promising outcomes of the robotic thyroidectomy approach, several limitations are found in this systematic review. Reporting and selection biases regarding the absence of randomized controlled trials during the past five years included in our study. Regional biases most operations are conducted in the far east more than in the western world. In addition, the choice of the procedure depends on the patient’s decision, accordingly and an explanation of the procedure and the associated risks must be explained to all the patients in detail in the informed consent [1]. 

Lastly, long-term investigations and follow-up studies with prospective randomized trials are essential adoption of the robotic technique in more specialized centers and hospitals around the world not only centered in the far east. With more learning and training courses for the surgeons because the decision of the approach depends largely on their skills and high volume of surgeries [8].

## Conclusions

Although all the robotic thyroidectomy approaches shared a longer operative time, they all showed better and more satisfying cosmetic results compared to the open conventional techniques. Also feasibility of lymph nodes dissection, the same hospital stay as the open thyroidectomy but lesser post-operative pain, swallowing discomfort, and voice changes compared to this latter technique. Although the advantages of these robotic thyroidectomy approaches, multiple limitations must be taken into consideration. For example, according to the American thyroid association, patients undergoing robotic thyroidectomy must be in a normal range of the body weight (except for the retro-auricular approach. In addition, the range size of the thyroid nodule must be within 3 to 4 cm, as it’s noted in a small number of studies.

Robotic thyroidectomy is a promising technique but is highly dependent on the surgeon's skills and the number of surgeries performed. In addition, the decision of the patient in choosing between the open and the robotic thyroidectomy is very important. Furthermore, these techniques are still regionally limited as the performance in the western countries and the United States is rare. In addition, learning centers all over the world must be considered aiming to increase the skills and performances of the surgeon, as more experiences reduce the operative time.

Finally, more retrospective reviews and studies concerning all robotic thyroidectomy techniques are required to be performed on a large number of patients of different gender and age and from multiple operative centers worldwide, with a long follow-up for early and late complications.
